# Angiotensin type 1 receptor antagonist losartan, reduces MPTP-induced degeneration of dopaminergic neurons in substantia nigra

**DOI:** 10.1186/1750-1326-2-1

**Published:** 2007-01-15

**Authors:** Tom N Grammatopoulos, Susan M Jones, Ferogh A Ahmadi, Brian R Hoover, Lawrence D Snell, Jesse Skoch, Vimal V Jhaveri, Andy M Poczobutt, James A Weyhenmeyer, W Michael Zawada

**Affiliations:** 1 Division of Clinical Pharmacology and Toxicology, Department of Medicine, Denver and Health Sciences Center, Denver, Colorado 80262, USA; 2 Neuroscience Program, Department of Medicine, Denver and Health Sciences Center, Denver, Colorado 80262, USA; 3 Department of Pharmacology, University of Colorado at Denver and Health Sciences Center, Denver, Colorado 80262, USA; 4 Department of Cell and Structural Biology, University of Illinois, Urbana, Illinois 61801, USA

## Abstract

**Background:**

Recent attention has focused on understanding the role of the brain-renin-angiotensin-system (RAS) in stroke and neurodegenerative diseases. Direct evidence of a role for the brain-RAS in Parkinson's disease (PD) comes from studies demonstrating the neuroprotective effect of RAS inhibitors in several neurotoxin based PD models. In this study, we show that an antagonist of the angiotensin II (Ang II) type 1 (AT_1_) receptor, losartan, protects dopaminergic (DA) neurons against 1-methyl-4-phenyl-1,2,3,6-tetrahydropyridine (MPTP) toxicity both in primary ventral mesencephalic (VM) cultures as well as in the substantia nigra pars compacta (SNpc) of C57BL/6 mice (Fig. 1).

**Results:**

In the presence of exogenous Ang II, losartan reduced MPP^+ ^(5 μM) induced DA neuronal loss by 72% *in vitro*. Mice challenged with MPTP showed a 62% reduction in the number of DA neurons in the SNpc and a 71% decrease in tyrosine hydroxylase (TH) immunostaining of the striatum, whereas daily treatment with losartan lessened MPTP-induced loss of DA neurons to 25% and reduced the decrease in striatal TH^+ ^immunostaining to 34% of control.

**Conclusion:**

Our study demonstrates that the brain-RAS plays an important neuroprotective role in the MPTP model of PD and points to AT_1 _receptor as a potential novel target for neuroprotection.

## Background

Parkinson's disease (PD) was originally described in 1817 by James Parkinson and since then there has been much progress in determining the etiology of the disease [1]. There is now clear evidence showing that the primary pathological feature of PD is the loss of dopaminergic (DA) neurons in the substantia nigra pars compacta (SNpc) [2]. Insights into the mechanisms responsible for PD have come from epidemiological studies and through animal models of DA neurodegeneration [3-5]. Treatment of rodents and non-human primates with neurotoxins such as 1-methyl-4-phenyl-1,2,3,6-tetrahydropyridine (MPTP), 6-hydroxydopamine [6,7] and rotenone [8] have helped us understand that oxidative stress, mitochondrial respiration dysfunction and protein aggregation are primary mediators of the dopaminergic neurodegeneration [9]. Even with some understanding of how DA neurons are lost in PD, there is still no effective therapy to halt or slow down the progression of the disease. Several clinical studies have promised the development of new therapies for prevention of dopaminergic neurodegeneration, such as through the use of resagiline, co-enzyme Q10, memantine and others [10]. However, additional research is needed to identify new molecular targets, which may help find ways to prevent or reduce the DA neuronal loss in PD. This study explores the hypothesis that the renin-angiotensin system (RAS) is a potential target for preventing the loss of DA neurons.

The renin-angiotensin system is best known for its role in regulating blood pressure, activation of sympathetic pathways, stimulation of vasopressin release, regulating drinking behavior and cerebral blood flow [11,12]. Only recently has it been discovered that all the required components of the RAS, such as renin, angiotensinogen, angiotensin converting enzyme (ACE), angiotensin II (Ang II) and the Ang II (AT) receptors, are present in the mammalian brain [13,14]. Ang II is the primary agonist of the RAS and has similar affinities for the two primary AT receptors, AT_1 _and AT_2_. The AT_1 _receptor was originally identified by the selective binding of a non-peptide receptor antagonist, 2-n-butyl-4-chloro-5-hydroxy-methyl-1-[(2'-(1H)-tetrazol-5-yl)biphenyl-4-yl)methyl]imidazol potassium salt (DuP 753, losartan), whereas the AT_2_, receptor was identified by the selective binding of a non-peptide receptor antagonist, PD123319 [15]. Both the AT_1 _and AT_2 _receptors are known to be members of the seven-transmembrane spanning G-protein coupled receptor (GPCR) superfamily [15]. AT_1 _receptor signaling activates kinases through a protein kinase C (PKC) pathway and AT_2 _receptor signaling activates phosphatases through a phospholipase-2 (PLA2) pathway. The two receptor types have been suggested to have opposing actions [16].

Recently, increased attention has focused on understanding the role of the brain-RAS in stroke and neurodegenerative diseases such as Alzheimer's disease and multiple sclerosis [17-24]. Several groups have proposed the possibility that the brain-RAS may also have a role in PD. One genetic study, suggests that polymorphisms in the gene encoding for ACE may be a risk factor for PD [25]. Examination of human postmortem brain tissues has shown a loss of both AT_1 _and AT_2 _receptor binding sites in the substantia nigra of PD patients, suggesting that PD-induced neurodegeneration may involve AT receptor expressing cells [18]. The brain-RAS is also involved in maintaining tyrosine hydroxylase (TH) transcription and catecholamine synthesis [26-30]. Recently, Ang II was shown to increase the number of DA neurons *in vitro*, via an action on the AT_2 _receptor [31]. More direct evidence of a role for the brain-RAS in PD comes from studies demonstrating the neuroprotective effects of ACE inhibitors in MPTP and 6-hydroxydopamine-treated rodents [32-35]. We have previously found that *in vitro *antagonism of the AT_1 _receptor with losartan, and subsequent activation of the AT_2 _receptor with exogenous Ang II, protects primary ventral mesencephalic DA neurons against the mitochondrial complex I inhibitor, rotenone [20]. In the current study, we demonstrate for the first time that the AT_1 _receptor antagonist, losartan, can protect DA neurons of the SNpc against MPTP-induced toxicity in C57BL/6 mice. These studies raise the possibility that AT_1 _receptor antagonism may be a novel method for preventing the loss of DA neurons.

**Figure 1:**
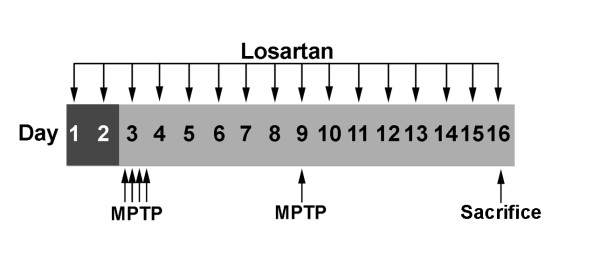
Timeline of the experimental design. Arrows point to when subcutaneous injections of losartan (90 mg/kg) and intraperitoneal injections of MPTP-hydrochloride (20 mg/kg) were administered and when the animals were sacrificed.

## Results

### Angiotensin II protects dopaminergic neurons in vitro from MPP^+ ^toxicity only in the presence of the AT_1 _receptor antagonist losartan

**Figure 2:**
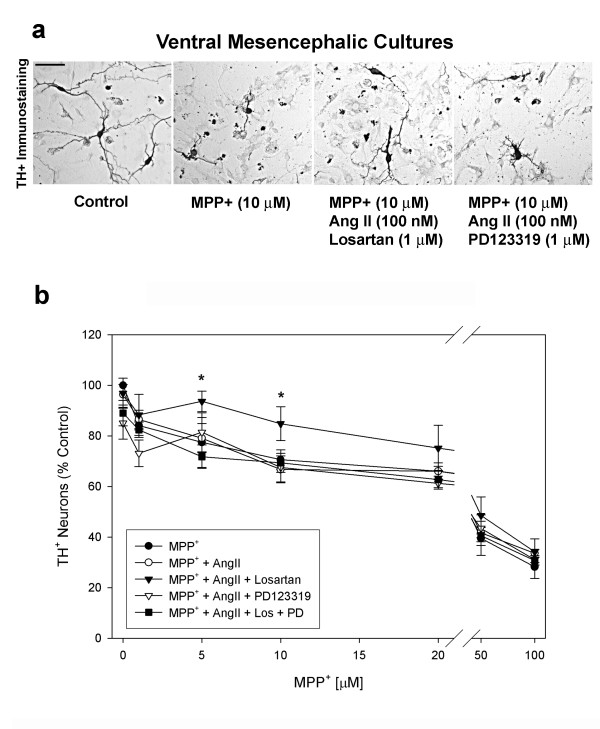
Angiotensin II protects DA neurons *in vitro *from MPP^+ ^toxicity only in the presence of the AT_1 _receptor antagonist losartan. (**a**) Microscopic view of TH^+ ^(DA) neurons in VM cultures in the presence of media or MPP^+ ^(10 μM) alone, MPP^+ ^with Ang II (100 nM), and MPP^+ ^with either losartan (1 μM) or PD123319 (1 μM). Scale bar equal to 50 μm. (**b**) Quantification of TH^+ ^neuron counts in VM cultures treated with increasing concentrations of MPP^+ ^(1–100 μM) in the presence or absence of Ang II (100 nM) and AT receptor antagonists, losartan (1 μM) (AT_1_R) and/or PD123319 (1 μM) (AT_2_R). Results are mean ± SEM. (n = 4–6). (*) Represents a significant difference (p ≤ 0.05, One-way ANOVA followed by Newman-Keuls post-hoc test) from MPP^+^.

To study the effects of the RAS on MPP^+^-induced neurotoxicity *in vitro*, primary rat E15 VM cultures were grown for 6 days and then treated with MPP^+ ^(1–100 μM) for 48 hrs. MPP^+^-treated cultures showed a dose-dependent loss of TH immunoreactive DA neurons, with a statistically significant loss of 15.9 ± 3% to 71.8 ± 6% over a dose range of 1 to 100 μM (Fig. 2).

In an effort to determine whether AT receptor activation affects the resistance of DA neurons to MPP^+ ^toxicity, VM cultures were treated with Ang II (100 nM) prior to the addition of MPP^+ ^(1–100 μM). Select VM cultures were also pretreated with the AT_1 _receptor antagonist losartan (1 μM) and/or the AT_2 _receptor antagonist PD123319 (1 μM), prior to the addition of Ang II (Fig. 2). TH^+ ^neuronal counts were determined 48 hrs after the addition of MPP^+^. MPP^+ ^dose response conditions were analyzed by one-way ANOVA followed by a Newman-Keuls post-hoc test. This analysis showed that MPP^+^-treated VM cultures pretreated with Ang II (100 nM), in the presence of losartan (1 μM), had a statistically significant reduction in DA neuronal loss when compared to MPP^+ ^exposed VM cultures pretreated with Ang II alone or in the presence of PD123319 (Fig. 2). VM cultures treated with Ang II and losartan showed a 71.6 ± 6% and 48.3 ± 10% reduction in MPP^+^-induced DA neuronal loss when compared to 5 μM and 10 μM MPP^+ ^alone treated VM cultures, respectively (Fig. 2). In combination, both AT receptor antagonists in the presence of Ang II (100 nM) did not significantly alter MPP^+ ^toxicity. Ang II alone or in the presence of PD123319, did not provide significant neuroprotection (Fig. 2). Both AT receptor antagonists, in the absence of exogenous Ang II, also did not affect MPP^+ ^toxicity (data not shown). In addition, VM cultures treated with Ang II (100 nM) and losartan (1 μM) 15 to 60 min after the addition of MPP^+ ^(10 μM) and assayed 48 hrs later, were not neuroprotected against MPP^+ ^(data not shown). This suggests that modification of downstream signaling of the angiotensin pathway is required prior to neurotoxin exposure.

### In vitro AT receptor expression profile in primary VM cultures

**Figure 3:**
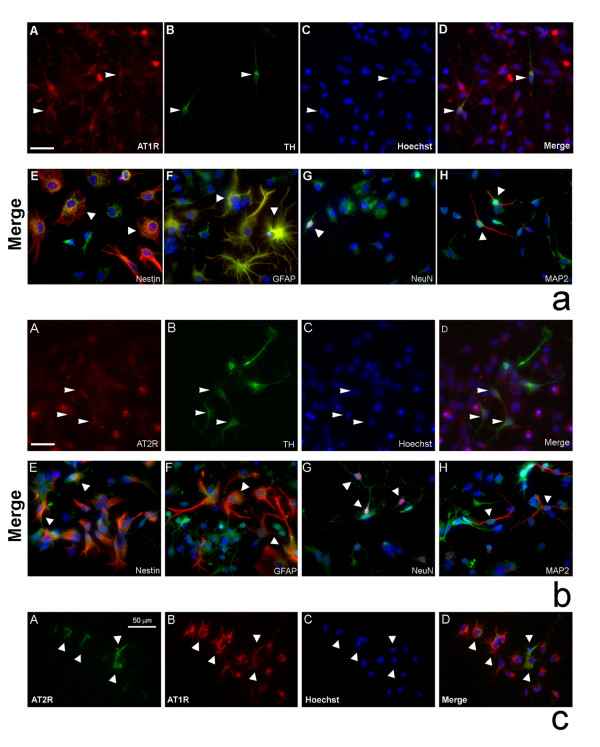
*In vitro *AT receptor expression profiles in primary rat VM cultures. (**a**) VM cultures were stained for TH (DA neurons) (green) (**A**), AT_1 _receptor (red) (**B**), and counter stained with Hoechst dye (blue nuclear stain) (**C**). Merged image of A-C (**D**). Panels (**E-H**) are merged images of AT_1 _receptor (green) co-labeling with either nestin (neural progenitors; red) (**E**), GFAP (astrocytes; red) (**F**), NeuN (neurons; red) (**G**), or MAP2 (neuronal marker; red) (**H**), counter stained with Hoechst dye (blue nuclear stain) and visualized under fluorescence microscopy (×400). Arrowheads indicate examples of cells of various phenotypes that contain AT_1 _receptors. Scale bar equal to 50 μm. (**b**) VM cultures were stained for TH (green) (**A**), AT_2 _receptor (red) (**B**), and counter stained with Hoechst dye (**C**). Merged image of A-C (**D**). Panels (**E-H**) are merged images of AT_2 _receptor (green) double stained with either nestin (red) (**E**), GFAP (red) (**F**), NeuN (red) (**G**), or MAP2 (red) (**H**), counter stained with Hoechst dye and visualized under fluorescence microscopy. Arrowheads point to various cell types positive for the AT_2 _receptor (×400). Scale bar equals 50 μm. (**c**) Panels (**A-D**) show co-expression of AT_1 _(red) (**B**) and AT_2 _(green) (**A**) receptors counter stained with Hoechst dye (**C**). Merged image of A-C (**D**). Arrowheads show examples of cells co-expressing both types of AT receptors.

Since downstream signaling of Ang II is dependent on specific AT receptor distribution, we began examining AT_1 _and AT_2 _receptor expression in DA neurons *in vitro*. While the majority (95%) of TH^+ ^neurons expressed the AT_1 _receptor using two different antibodies (Abcam and Santa Cruz) (Fig. 3a), only 65% of the TH^+ ^neurons expressed the AT_2 _receptor (detectable only with Abcam antibody) (Fig. 3b). Because of the difference in AT_1 _and AT_2 _receptor expression in DA neurons, the AT receptor profiles of other VM culture cell types were subsequently examined.

AT_1 _receptors were co-expressed in cells immunopositive for nestin (neural progenitors), GFAP (astrocytes), NeuN and MAP2 (neurons) (Fig. 3a). AT_2 _receptors were expressed in nestin, GFAP, NeuN and MAP2 stained cells (Fig. 3b). Approximately half of all cells showed double labeling for both AT receptors (Fig. 3c).

**Figure 4:**
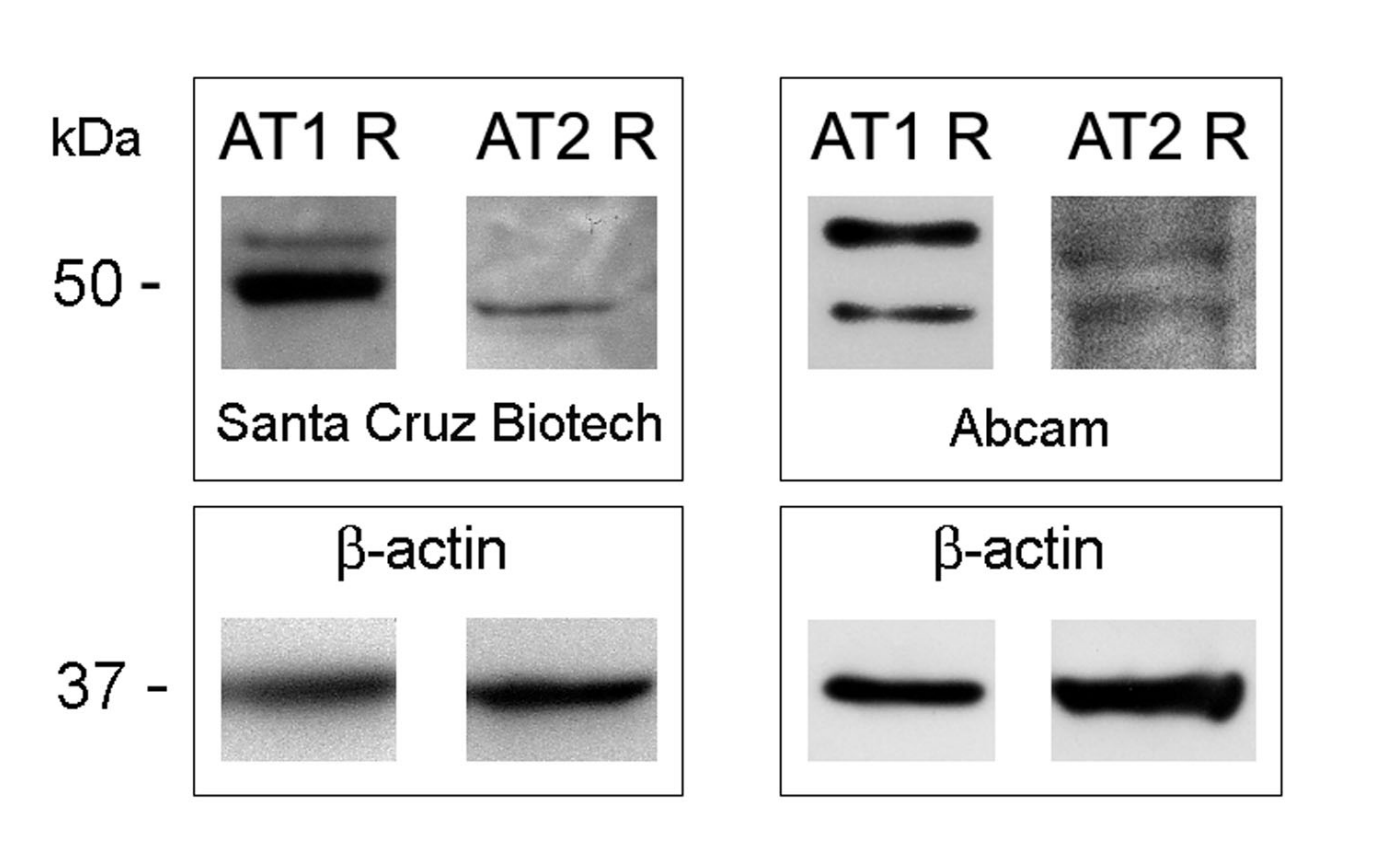
Western immunoblot detection of the AT receptors in N27 dopaminergic cell line using either the Abcam or the Santa Cruz antibodies. The AT_1 _receptor is detected as two proteins representing the AT_1A _(43 kDa) and AT_1B _(53 kDa) encoded by two different genes. The AT_2 _is detected at 43 kDa.

In an effort to determine specificity of the antibodies used for the detection of the AT_1 _and AT_2 _receptors we performed western immunoblot analysis of these receptors in N27 dopaminergic cells, which were derived from immortalized E12 VM. As seen in figure 4, both antibodies detect the AT_1A _and the AT_1B _and AT_2 _receptors in these dopaminergic cells.

### In vivo AT receptor expression profile in dopaminergic neurons of the substantia nigra pars compacta (SNpc)

**Figure 5:**
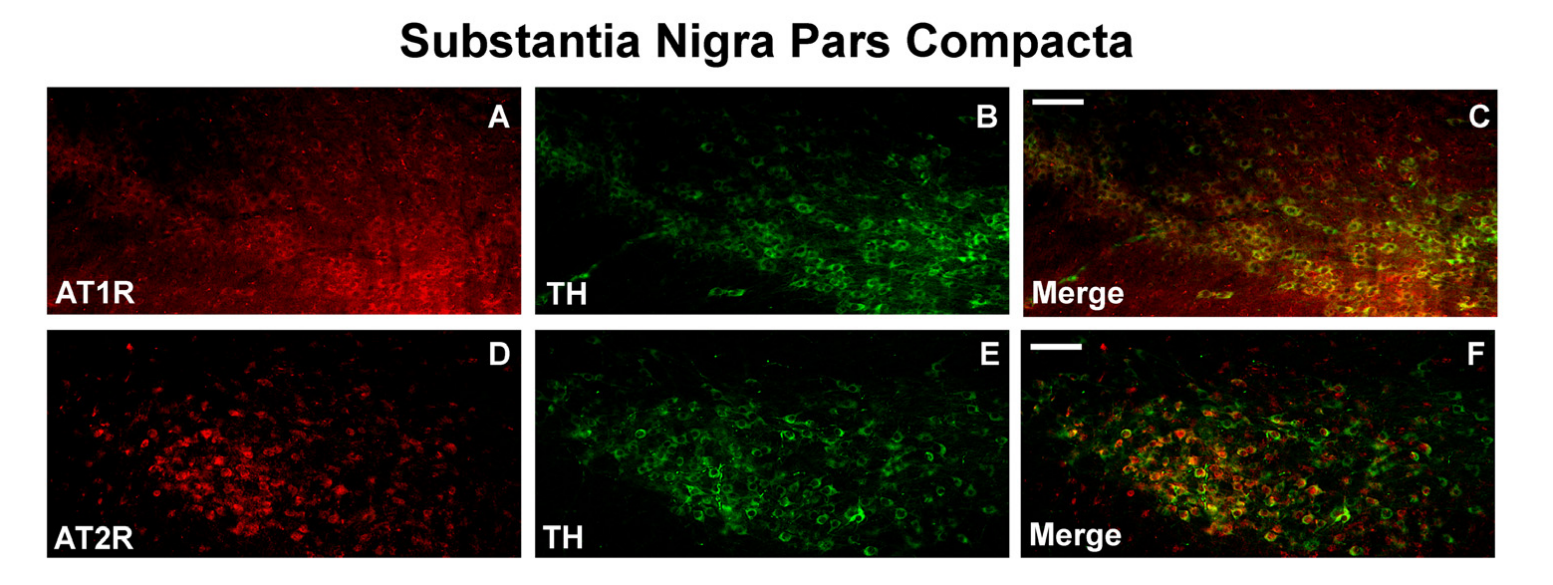
*In vivo *AT receptor profile of DA neurons in the substantia nigra pars compacta (SNpc) of adult C57BL/6 male mice. Coronal sections (40 μm) were stained for (A) AT_1 _receptor (red) or (D) AT_2 _receptor (red), (B, E) TH (DA neurons) (green) and (C, F) represent merged images. Scale bar equals to 200 μm.

**Figure 6:**
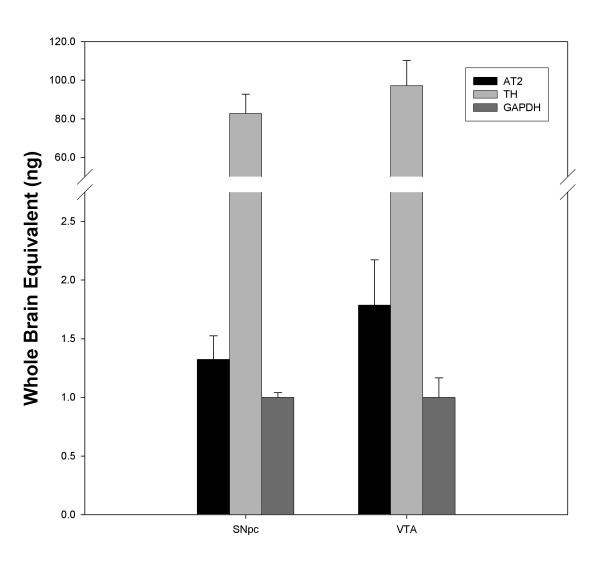
Detection of Agtr2, TH and GADH mRNAs by real time RT-PCR in TH^+ ^neurons of the SNpc and VTA. Relative concentrations were determined using a standard curve of known concentrations of whole brain cDNAs.

To better understand the role of the brain-RAS in preventing DA neuronal loss *in vivo*, we determined the AT receptor expression profile of TH^+ ^cells of the SNpc of adult male C57BL/6 mice by immunohistochemistry. As seen in figure 5, TH^+ ^neurons in the intact SNpc expressed a similar AT receptor profile to that found in VM cultures, with the majority of TH^+ ^cells expressing the AT_1 _receptor (95%), but the AT_2 _receptor was expressed in fewer SNpc dopamine neurons (45%); detectable only with the Abcam antibody (Fig. 5). Because of the inherently semi-quantitative nature of the immunohistochemical analysis, we have also employed an alternative technique for assaying the AT_2 _receptor expression by determining the levels of Agtr2 mRNA by real-time RT-PCR, in TH^+ ^neurons of the SNpc and ventral tegmental area (VTA), isolated by laser capture microdissection (LCM). Low levels of Agtr2 mRNA were detected in these cells and there was no significant difference between TH^+ ^neurons from the SNpc and the VTA (Fig. 6). Animal and cell culture treatments with MPTP or MPP^+ ^did not change AT receptor immunoreactivity to any detectable level (data not shown). In addition, we did not observe any differences in the AT receptor profile between TH^+ ^neurons of the SNpc and TH^+ ^neurons of the VTA (data not shown), indicating that previously reported lower susceptibility to MPTP of the TH^+ ^neurons from the VTA is not likely due to a difference in AT receptor distribution.

### Losartan protects SNpc dopaminergic neurons from MPTP toxicity

**Figure 7:**
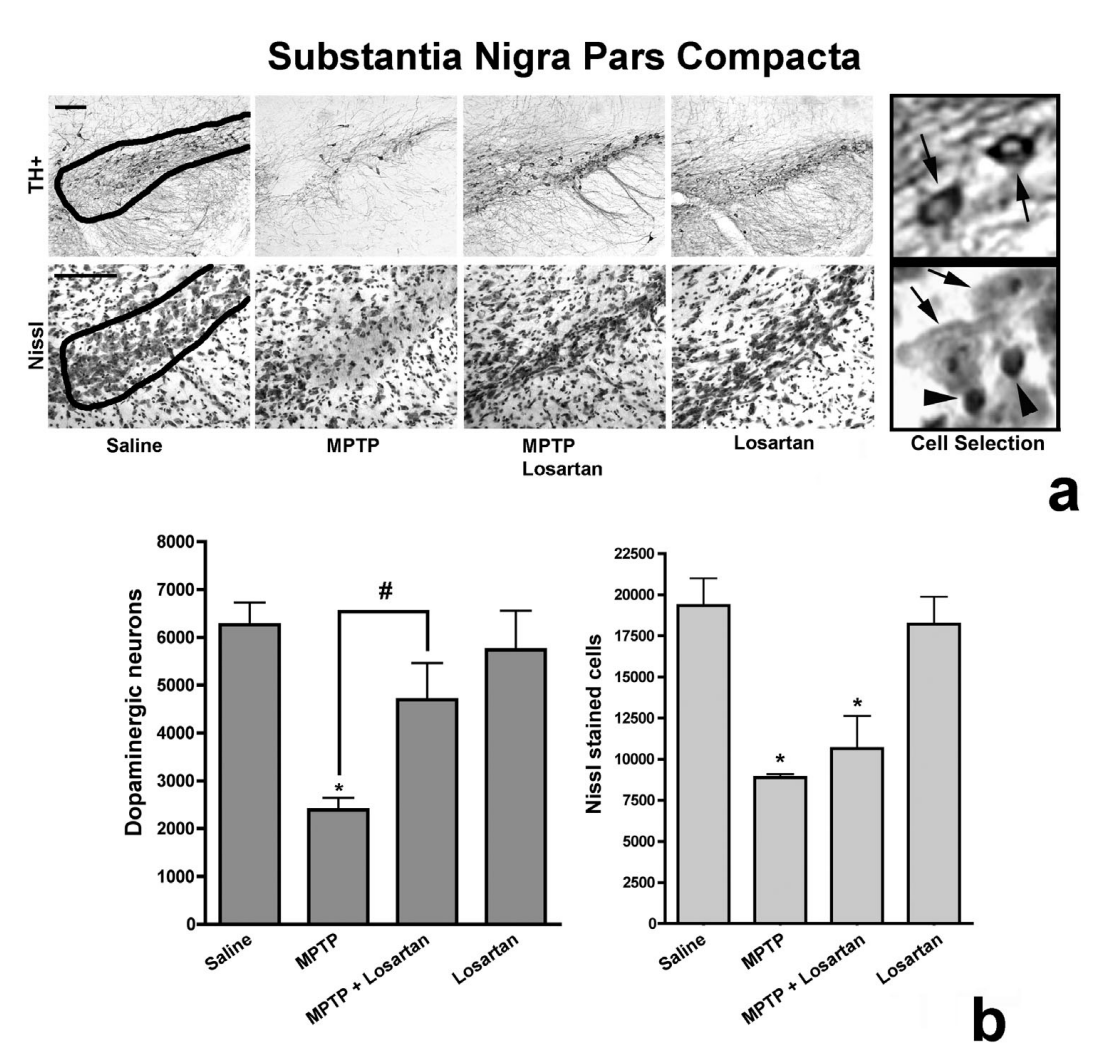
Losartan protects DA neurons of the SNpc from MPTP toxicity. (**a**) TH immunoreactive neurons and Nissl stained cells in the SNpc from mice treated with saline, MPTP, MPTP + losartan or losartan alone. Scale bars equal to 100 μm. Saline panel shows a representative contour selection of the A9 region. The Cell Selection panel shows an example of counted TH^+ ^neurons (indicated with arrows) or Nissl-stained cells with large nuclei (indicated with arrows) and excluded Nissl-stained cells with small nuclei (indicated with arrowheads). (**b**) Quantification of the number of TH^+ ^neurons and Nissl stained cells in the SNpc, revealed a significant decrease in the number TH^+ ^neurons in MPTP treated mice when compared to saline control treated animals. MPTP-injected mice treated daily with losartan significantly decreased the MPTP-induced loss of TH^+ ^neurons in the SNpc. Significance is indicated by (#) when compared to MPTP-alone injected mice and (*) when compared to saline-alone injected mice (p ≤ 0.05, One-way ANOVA followed by Newman-Keuls post-hoc test). Data are represented as mean ± SEM, n = 4–5 for TH immunostain and n = 3 for Nissl stain.

A severe loss (61.8 ± 10%) of DA neurons was observed in the SNpc of MPTP-treated mice compared to control mice treated with saline (Fig. 7). By contrast, the number of DA neurons lost in MPTP-treated mice receiving losartan was only 25.0 ± 16%, when compared to saline-treated mice. These results show that pretreatment and daily dosing with losartan can reduce MPTP-induced DA neuronal loss in the SNpc by 60% (p < 0.05). In an effort to confirm that the injected MPTP regimen induced DA neuronal loss and not just a down-regulation of TH immunoreactivity, we determined the total number of cells in the SNpc by Nissl staining. Nissl staining of the SNpc revealed a total cell loss of 54.1 ± 1% in MPTP-treated mice, demonstrating that the MPTP regimen resulted in DA neuronal cell loss (Fig. 7). MPTP-treated mice receiving losartan had a 44.9 ± 10% loss of total cells, which was not statistically different from the cell loss observed in mice receiving only MPTP. In mice treated with losartan alone, the numbers of DA neurons or Nissl-stained cells remained unaltered when compared to saline control mice (Fig. 7). Our results show that the AT_1 _receptor antagonist, losartan, reduces MPTP-induced DA neuronal loss in the SNpc of C57BL/6 mice.

### Losartan protects striatal nerve terminals from MPTP-induced toxicity

**Figure 8:**
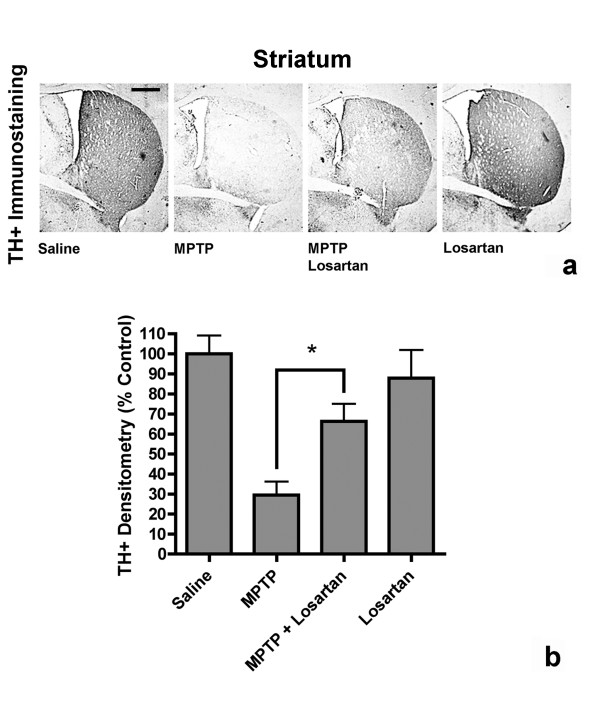
Losartan prevents MPTP-induced denervation of the striatum. (a) Coronal sections of TH immunoreactivity in the striatum of mice treated with saline, MPTP, MPTP + losartan or losartan alone. Scale bars equal to 500 μm. (b) Quantification of striatal densities, showing a significant decrease in the percent of TH^+ ^immunoreactivity in MPTP-treated mice. MPTP-injected mice treated daily with losartan had significantly higher levels of TH^+ ^immunoreactivity in the striatum when compared to MPTP alone-injected mice (*, p ≤ 0.05, One-way ANOVA followed by Newman-Keuls post-hoc test). Data are represented as mean ± SEM, (n = 4–5).

Significant differences between treated animal groups were also observed when measuring striatal TH^+ ^immunostaining densities. Mice treated with MPTP showed a significant 70.6 ± 7% decrease in striatal TH^+ ^staining when compared to saline control animals (Fig. 8). Daily treatment with losartan significantly reduced the loss of striatal TH immunoreactivity in the striatum of MPTP-treated mice by 53.2 ± 8% when compared to MPTP-alone treated mice (Fig. 8). Animals treated with losartan alone showed no significant change in TH immunoreactivity when compared to saline control animals (Fig. 8). Our results demonstrate that inhibition of the AT_1 _receptor with losartan significantly reduces MPTP-induced denervation of striatal nerve terminals.

### Losartan does not interfere with transport of MPTP into the brain and its conversion to MPP^+ ^in C57BL/6 mice

The possibility that losartan may interfere with brain uptake or metabolism of MPTP was examined in the SN and striatum of mice injected with MPTP. Sixty minutes after the last MPTP injection, the SN and striatum were dissected and MPP^+ ^levels were determined through HPLC. No differences in nigral and striatal MPP^+ ^levels were observed regardless of whether MPTP-injected mice were treated with losartan or not (Table 1). These findings highlight the fact that losartan does not interfere with brain uptake or metabolism of MPTP.

**Table 1:** Losartan does not interfere with the metabolism and uptake of MPTP.

(a) MPP^+ ^levels in the striatum and substantia nigra of C57BL/6 mice		
Treated Groups	Striatum	Substantia nigra
MPTP	0.27 ± 0.03 μg MPP^+^/g tissue	0.28 ± 0.12 μg MPP^+^/g tissue
MPTP+ Losartan	0.26+0.04 μg MPP^+^/g tissue	0.22 ± 0.05 μg MPP^+^/g tissue

(b) Dopamine uptake in ventral mesencephalic cultures		

Control	68.1 ± 11.2 fmol/sec	
Losartan (1 μM)	72.7 ± 7.9 fmol/sec	
Losartan (10 μM)	76.2 ± 14.5 fmol/sec	

(a) MPP^+ ^levels in the striatum and substantia nigra of C57BL/6 mice were measured by HPLC analysis 60 min after MPTP injection regimen and were compared to MPTP injected mice pretreated with the AT_1 _receptor antagonist losartan (n = 3). (b) Dopamine uptake levels in VM cultures treated with media control, 1 and 10 μM losartan (n = 3).

### Losartan does not interfere with dopamine uptake in VM cultures

To rule out the possibility that losartan-mediated neuroprotection was a result of inhibition of the dopamine transporter (DAT) and subsequent reduction of MPP^+ ^entry into DA neurons, VM cultures were treated with losartan (1 and 10 μM) and DA uptake levels were determined. Losartan (1 and 10 μM) did not alter DA uptake in VM cultures (Table 1). This demonstrates that losartan does not interfere with the function of the DAT.

### Losartan reduces arterial blood pressure in C57BL/6 mice

In an effort to determine if losartan reduces blood pressure (BP) levels in C56BL/6 male mice, we took arterial BP measurements of mice treated with saline or losartan. In order to maintain constant BP levels, losartan was administered in drinking water at a dose of 0.18 mg/mL. Table 2 shows the systolic and diastolic measurements after this dosing regimen. Losartan alone reduced mean BP by 24 ± 3% when compared to saline treated controls (Table 2).

**Table 2:** Losartan reduces arterial blood pressure in C57BL/6 mice.

**Systolic**		
Treatment	Saline	Losartan

Mean	124.28	96.43
Std. error of mean (SEM)	3.53	3.45
Sample size (N)	39	43

**Diastolic**		

Treatment	Saline	Losartan

Mean	101.24	75.31
Std. error of mean (SEM)	4.68	3.21
Sample size (N)	33	38

Losartan (0.18 mg/mL) was administered through the drinking water and tail artery blood pressures were recorded non-invasively from awake animals. Data are represented as mean ± SEM, (n = 4 animals, >32 readings per animal).

## Discussion

The primary goal of this study was to determine if manipulation of brain-RAS with the AT_1 _receptor antagonist, losartan, could prevent the DA neuronal loss caused by the parkinsonism-inducing neurotoxin, MPTP. Losartan in the presence of exogenous Ang II *in vitro*, was able to significantly reduce the loss of DA neurons induced by MPP^+^. These results are in agreement with our earlier findings demonstrating that antagonism of the AT_1 _receptor with losartan could protect cultured DA neurons from rotenone toxicity [20]. In the current study, we also showed that pretreatment of MPTP-lesioned adult C57BL/6 mice with losartan significantly reduced the loss of DA neurons in the SNpc as well as partially spared striatal TH^+ ^terminals.

The brain-RAS has already been identified to have a potential beneficial and therapeutic value as a target against stroke. Several recent studies have shown that peripheral treatment with AT_1 _receptor antagonists is protective against ischemia and reduce the cortical volume of the ischemic lesion [36,37]. We have recently demonstrated that Ang II, through the actions of the AT_2 _receptor, protects primary cortical neuronal cultures from hypoxic injury [19,38]. In addition, we also demonstrated that the AT_2 _receptor-mediated neuroprotection is dependent on the delayed rectifier K^+ ^channel, Na^+^/Ca^+2 ^exchanger and Na^+^/K^+^ATPase [39]. And although we have not yet conclusively identified the mechanism(s) of Ang II-mediated DA neuroprotection, our previous work, as well as studies by others, provide some insight into potential mechanisms.

Several groups have suggested a role for the brain-RAS in PD. A genetic study has implicated polymorphisms of the angiotensin-converting enzyme (ACE) gene as a risk factor for PD [25]. In addition, postmortem studies have shown the loss of both AT_1 _and AT_2 _receptor binding sites from the SN in PD patients, suggesting that PD-induced neurodegeneration may involve AT receptor expressing cells [18]. The brain-RAS is also involved in maintaining TH transcription and catecholamine synthesis [26-30]. In this study, we observed that almost all DA neurons express the AT_1 _receptor and to a lesser extent they express the AT_2 _receptor, both *in vitro *and *in vivo*. Because of the low level of AT_2 _receptor immunostaining, we confirmed the presence of Atgr2 mRNA in TH^+ ^neurons by real-time RT-PCR. The immunohistochemical analysis also revealed that all other cell types examined express both AT receptor subtypes *in vitro*. These results suggest that the observed *in vivo *neuroprotection may be mediated through the antagonism of the AT_1 _receptor and potentially the subsequent activation of the AT_2 _receptor by Ang II endogenously present in the brain. These data are in agreement with our previous findings which suggest a negative role for the AT_1 _receptor and a neuroprotective role for the AT_2 _receptor which under normal conditions is masked by the opposing role of the AT_1 _receptor [20]. An alternative hypothesis is that because DA neurons have low levels of the AT_2 _receptor, it is possible that the observed neuroprotection may be due to the inhibition of downstream signaling of the AT_1 _receptors directly in DA neurons and the preferential activation of AT_2 _receptors in supporting cells, which then indirectly protect the neurons. In support of the indirect theory, astrocytes are known to produce factors such as glial cell line-derived neurotrophic factor (GDNF) and mesencephalic astrocytes-derived neurotrophic factor (MANF), two highly potent neurotrophic factors for DA neurons [40,41]. It should be noted that because losartan did reduce the mean arterial BP in mice, we could not exclude the possibility that the reduction in BP may be contributing to losartan's neuroprotective actions *in vivo*. Further research is needed to answer many of these questions.

A recent observation by Rodriguez-Pallares et al [31] describes that developing rat DA neurons derived from E14 VM express the AT_2 _receptor. Our study extends these findings and quantitates the proportion of DA neurons that express the AT_2 _receptor, both in VM cultures (65%) and in the SNpc of C57BL/6 mice (45%). We also now report expression of the AT_2 _receptor on nestin positive VM precursors. Our observation of abundance of the AT_2 _receptor on immature cells is corroborated by others, who have reported that during development AT_2 _receptor expression levels are high in the brain but significantly decrease during adulthood [42]. More direct evidence for a role of the brain-RAS in PD comes from studies examining the neuroprotective effects of ACE inhibitors in 6-hydroxydopamine and MPTP-induced neurotoxicity rodent models [32-35]. ACE inhibitors prevent the formation of Ang II from the decapeptide Ang I, which results in indiscriminate reduction in signaling at all AT receptors, including a potential beneficial AT_2 _effect. Our study demonstrates that similar neuroprotection can be obtained by selective antagonism of the AT_1 _receptor, in the absence of disruption of Ang II generation and its activity at other AT receptors. One possible mechanism for the neuroprotective effects resulting from inhibiting the AT_1 _receptor might relate to oxyradical generation. Activation of the AT_1 _receptor can lead to upregulation of NADPH oxidase, a mechanism for generation of superoxide and elevation of oxidative stress [51].

In addition, the AT_1 _receptor has been shown to induce an upregulation of TH, which may result in higher levels of DA [26-28]. Studies have suggested that elevated DA levels may lead to DA quinones, which can mediate oxidative stress. Additional processes that might contribute to susceptibility of DA neurons in PD include: (1) toxicity of tetrahydrobiopterin (an obligatory cofactor for TH) via increased DA production and the cofactor's autooxidation [52] and (2) increasing catecholamine concentrations via overexpression of α-synuclein and resulting disruption of vesicular pH that perturbs the ability of vesicles to store neurotransmitters [53]. Others however argue that elevated DA actually inhibits formation of α-synuclein aggregates [54] and that depletion of DA *in vivo *does not protect from acute toxicity of MPTP [55]. Taken together, these findings have to be interpreted in the context of variations in the models, neurotransmitter concentrations, and time scales, all of which affect the outcome. Nonetheless, most of these studies point to a likely central role for DA in the pathogenesis of PD.

While it may be beneficial to prevent AT_1 _receptor signaling through the use of ACE inhibitors, the reduction of Ang II in the brain will also prevent the neuroprotective actions of AT_2 _receptor-mediated signaling. The beneficial attributes of the AT_2 _receptor can be seen in studies describing how the AT_2 _receptor counteracts the actions of the AT_1 _receptor by means of its downstream signaling cascades or provide neuroprotection against mitochondrial toxins when unopposed by the AT_1 _receptor through the use of specific receptor antagonists targeting the AT_1 _receptor [15,19,38,39]. In addition, recent studies have also shown a relationship between metabolites of Ang II, such as angiotensin IV, which is believed to be the agonist for the AT_4 _receptor and is suggested to have a role in memory [24,44-47].

## Conclusion

In this study, we demonstrate that antagonism of the AT_1 _receptor can protect dopaminergic neurons from a neurotoxin based *in vivo *PD model. While we are actively investigating the role of the brain-RAS in PD, the angiotensin system may have a role in other neurodegenerative diseases such as Alzheimer's disease, multiple sclerosis, and Huntington's disease [22,48]. Additional research will be needed to completely understand the roles of this system in the brain.

## Methods

### Ventral mesencephalic culture

Rat ventral mesencephalic (VM) tissues from E15 Sprague Dawley embryos were dissected into Ca^+2^/Mg^+2 ^– free Hank's buffered salt solution (HBSS), mechanically dispersed and centrifuged [49]. The cells were resuspended in F12 medium with 10% fetal bovine serum, 2 mM L-glutamine, 100 U/ml penicillin, and 100 μg/ml streptomycin (Sigma). Viability was determined using trypan blue exclusion. Cells were seeded at a density of 6 × 10^4 ^viable cells/cm^2 ^on polyethylenimine (1 μg/ml) pre-coated 96- and 12-well culture plates or 8-chambered glass slides and incubated for 6 days at 5% CO_2_/37°C. Half of the medium was changed every 3–4 days. After 6 days of growth, primary VM cultures consist of approximately 55% neurons, 45% type 1 astrocytes and <1% other cell types, as determined by immunohistochemical analysis [50]. Dopaminergic (TH^+^) neurons represented 2–5% of total cells in VM cultures.

### Ventral mesencephalic culture treatments

VM cultures were exposed to the parkinsonism-inducing neurotoxin 1-methyl-4-phenylpyridinium (MPP^+^) (1–100 μM) (Sigma) for 48 hr at 37°C and then processed for analysis. In Ang II-treated cultures, the octapeptide (1–100 nM) (Sigma) was added either 30 min prior or 15, 30 or 60 min after MPP^+ ^treatment. In an effort to determine AT receptor subtype specificity, VM cultures were pretreated with 2-n-Butyl-4-chloro-5-hydroxy-methyl-1-[(2'-(1H)-tetrazol-5-yl)biph enyl-4-yl)methyl]imidazol potassium salt (DuP 753, losartan) (1 μM) (AT_1 _receptor antagonist) (Du Pont/Merck, Wilmington, DE) or PD123319 (1 μM) (AT_2 _receptor antagonist) (Sigma) or both, 15 min prior to the addition of Ang II [19,20]. Cells were exposed to the treatment agents for the duration of the experiment. VM cultures were then washed with PBS and fixed by the addition of 4% paraformaldehyde until analyzed.

### Animals and treatments

Eight-week-old male C57BL/6 mice (Charles River Laboratories, Wilmington, MA) were used. Mice (n = 4–5 per group) received subcutaneous (200 μl) injections of 2-n-Butyl-4-chloro-5-hydroxy-methyl-1-[(2'-(1H)-tetrazol-5-yl)biphenyl-4-yl)methyl]imidazol potassium salt (DuP 753, losartan) (90 mg/kg) (100 mg tablets, Du Pont/Merck, Wilmington, DE; dissolved in saline and filtered through a 0.2 μm filter) daily for 16 days, beginning two days prior to the first MPTP injection (Fig. 1). It is expected that the current dosing of losartan should result in greater than 60% inhibition of AT_1 _receptors in the brain [51]. Control animals received saline injections. Losartan and saline control treated mice received four intraperitoneal injections of MPTP-hydrochloride (20 mg/kg of free base; Sigma) in saline at 2-hour intervals on day 3 followed by a single intraperitoneal injection of MPTP-hydrochloride (20 mg/kg) on day 9, and were sacrificed 7 days after the last MPTP injection [52]. Control mice received saline injections. This protocol is in accordance with the NIH guidelines for use of live animals and was approved by the Institutional Animal Care and Use Committee of the University of Colorado at Denver and Health Sciences Center.

### Immunohistochemical analysis

For immunohistochemical analysis, animals were perfused with 20 ml of saline followed by 20 ml of freshly made 4% paraformaldehyde. Brains were then removed and stored in 4% paraformaldehyde until analyzed. Fixed VM cultures or 40 μm brain sections were incubated with an appropriate primary antibody overnight at 4°C, followed by 1 hr incubation with a secondary antibody at room temperature. To identify DA neurons, a polyclonal (rabbit) anti-TH antibody (1:500) (Pel-Freez, Rogers, AR) was used. TH^+ ^cells were visualized by nickel-enhanced DAB staining (Pierce) using a Vectastain kit (Vector laboratories, Burlingame, CA) or by immunofluorescence using an appropriate secondary antibody. Different cell types in the cultures were identified with a polyclonal (mouse) anti-glial fibrillary acidic protein (GFAP) (1:200) for astrocytes, a polyclonal (mouse) anti-nestin (1:400) for neural progenitors, and a polyclonal (mouse) anti-microtubule-associated protein 2 (MAP2) (1:200) or a polyclonal (mouse) anti-neuronal nuclear protein (NeuN) (1:200) for neuronal cell types (Chemicon, Temecula, CA). To determine AT receptor subtypes, a commercially available polyclonal (rabbit) anti-AT_1 _(N-10) (1:50) (Santa Cruz Biotechnology, Inc), polyclonal (goat) anti-AT_2 _(H-143) (1:50) (Santa Cruz Biotechnology, Inc), polyclonal anti-AT_1_(rabbit) (18801) (1:200) (Abcam), polyclonal (rabbit) anti-AT_2 _(C-18) (1:100) (Santa Cruz Biotechnology, Inc) or a polyclonal anti-AT_2 _(rabbit) (19134) (1:200) (Abcam) antibodies were used [19,20]. Secondary antibodies used were Alexa 488-conjugated (donkey) anti-goat IgG (1:1000), Alexa 488-conjugated (donkey) anti-rabbit IgG (1:1000), Alexa 594-conjugated (donkey) anti-rabbit IgG (1:1000) and Alexa 594-conjugated (donkey) anti-mouse IgG (1:1000) (Molecular Probes, Eugene, OR). Total cells were visualized by Hoechst stained nuclei. Double and triple staining was done consecutively. Stained cultures were visualized by light and/or fluorescence microscopy using Zeiss or Nikon microscopes. For AT receptor profile counts *in vitro*, a total of 700 TH^+ ^cells co-labeled for AT_1 _or AT_2 _receptor were counted from a minimum of 3 fields. For the *in vivo *AT receptor profile, TH^+ ^cells co-labeled for AT_1 _or AT_2 _receptor were counted from the SNpc of 3 coronal sections. To determine the number of total cells in the SNpc, selected sections were defatted, rehydrated through descending alcohol concentrations, stained in cresyl violet acetate for 1 minute followed by acetic formalin, then dehydrated and cleared in xylene before coverslipping in Permount. Quantification of VM culture TH^+ ^cells' survival was done by a blinded observer who counted all the positively stained cells having a distinct nucleus and visible neurites in the entire well. Each experimental condition *in vitro *represents 4–6 independent experiments.

### Measurements of striatal MPP^+ ^levels

HPLC with UV detection (wavelength = 295 nm) was used to measure striatal MPP^+ ^levels [56]. Mice were injected subcutaneously (n = 3) daily with losartan (90 mg/kg) or saline for 3 days. On day 3, mice were injected 4 times every 2 hours intraperitoneally with saline or MPTP-hydrochloride (20 mg/kg). Sixty minutes after the last MPTP injection mice were sacrificed and the brains were immediately removed. The substantia nigra (SN) and striatum were dissected out on ice and rapidly frozen on dry ice and stored at -80°C until analysis. On the day of the assay, SN and striatum were prepared by sonicating the tissue samples in 9% (wt/vol) of 5% trichloroacetic acid (Sigma) containing 5 μg/ml of 4-phenylpyridine (Sigma), as an internal standard. After centrifugation, 50 μl of supernatant were injected onto a cation-exchange C18 column (Alltech). The mobile phase consisted of 89% (v/v) 50 mM KH_2_PO_4 _(pH 3 adjusted with H_3_PO_4_) and 11% (v/v) acetonitrile. The flow rate was 1.5 ml/min.

### Dopamine uptake measurements

Dissociated mesencephalic neurons were grown as described above. The neurons were rinsed and then assayed at 37°C in Krebs-Ringer HEPES buffer (KRH; 120 mM NaCl, 4.7 mM KCl, 2.2 mM CaCl_2_, 1.2 mM Mg SO_4_, 1.2 mM KH_2_PO_4_, 10 mM glucose, 10 mM HEPES, pH 7.4) supplemented with 10 μM pargyline, 10 μM ascorbic acid, and 10 μM catechol. Assays (1 ml) included 100 nM [^3^H] DA. Nonspecific [^3^H] DA accumulation was determined in the presence of 1 mM (-) cocaine hydrochloride. After 10 min of incubation at 37°C, uptake was terminated by quickly washing the neurons three times with 1 ml of ice-cold KRH. Neurons were then solubilized in 0.5 ml of 3% trichloroacetic acid for 60 min with gentle shaking. Accumulated [^3^H] DA was determined by liquid scintillation counting (n = 3) [57].

### Animal blood pressure measurements

Losartan (0.18 mg/mL) was administered through the drinking water and tail artery blood pressures were recorded non-invasively from awake animals. Animals were placed in a restraining tube heated to 37°C, and a pressure cuff and piezoelectric transducer were secured to the rostral base of the tail (PowerLab, AD Instruments). Animals were conditioned for ten to fifteen minutes by inflating the pressure cuff to 200 mmHg once every minute. Post-conditioning pulse and cuff pressure recordings were simultaneously acquired for up to 45 min at one reading per minute. Motion artifact detected by the pulse transducer during cuff deflation served as grounds for elimination of data points. Systolic and diastolic pressures were determined offline (Chart, AD Instruments). Systolic pressure was defined as cuff pressure upon first detection of pulse during deflation. Diastolic pressure was defined as cuff pressure when the pulse reading resumed normal amplitude (n = 4, >32 readings per animal).

### Stereological analysis of TH^+ ^neurons and Nissl-stained cells in the SNpc

Substantia nigra sections were immunostained for TH or stained with cresyl violet as described above. The SN in C57BL/6 mice measures approximately 1.7 mm in rostrocaudal extent. Based on a comparative mouse brain atlas [58], the SN is completely contained between the coronal planes defined between -2.40 mm and -4.20 mm from bregma. A total of 54 (40 μm) sections were cut on a cryostat and separated into three section pools using every third section per pool. The three section pools contained the entire SN. Due to tissue shrinkage the actual thickness of fixed and stained tissue was on average 21 μm. TH-immunoreactive SNpc neurons were estimated using an unbiased stereological method according to the optical fractionator principle [59-61] using a Leica DMRB microscope with motorized stage running BioQuant Nova Prime Revision 3.0 (BioQuant Image Analysis Corporation, Nashville, TN) on a 75 μm × 75 μm grid with a dissector size of 50 μm × 50 μm. The contour outlines and landmarks of the SNpc, which comprise the A9 cell group, were drawn on an image captured with a 5× objective. Using a 100× objective and starting at the top of the section with the top plane of the cells in focus, the z-plane guards were determined by excluding 4 μm from the surfaces and only the TH^+ ^profiles that came into focus within the counting frame thickness (13 μm) between the guard zones were counted. Care was taken to ensure that the top and bottom forbidden planes were not included in the analysis. Adjacent sections were stained with cresyl violet acetate and cells in the SNpc were counted in a similar manner as with the TH^+ ^immunostained sections, with the exception that large nuclear Nissl stained cells were counted instead of TH^+ ^cells. Because Nissl staining was done separately from TH immunostaining, it is possible that non-dopaminergic neurons and glia were also included in the total cell counts. Because of the careful demarcation of the SNpc (Fig. 7a), the majority of large nuclear Nissl stained cells in the selected area are likely to be neurons of the A9 cell group. All stereological counts were done blindly to the treatment conditions. A coefficient of error (CE) of <0.02 was accepted (Table 3), for TH (n = 4–5 animals/group) and Nissl stained cells (n = 3 animals/group). Counts are represented as mean ± SEM.

**Table 3:** TH^+ ^neuronal counts and coefficient of variance (CV) values for all animal groups.

**Treatment**	**Saline**		**MPTP**		**MPTP+Los**		**Losartan**	
**Animal**	**Neurons**	**CV**	**Neurons**	**CV**	**Neurons**	**CV**	**Neurons**	**CV**

1	6749	0.08	3299	0.12	4028	0.09	3728	0.14
2	6138	0.06	2237	0.19	2837	0.06	5714	0.11
3	7389	0.09	2322	0.14	6144	0.10	7766	0.09
4	4591	0.07	1719	0.08	5762	0.07	5723	0.11
5	6446	0.08	2373	0.12				

### Striatal TH^+ ^staining densitometry

Striatal sections were stained for TH as described above. Digital images were taken of each striatal section (3 sections per animal counting both the left and right striatum, n = 4–5 animals/group) using a Nikon Eclipse 800 microscope and a CCD camera with Spot software. TH^+ ^staining densities were then analyzed using Adobe Photoshop software. Cortical areas (background) densitometry readings were subtracted from the measured densitometry of striatal areas. Measurements are represented as percent of control densities from saline-treated animals and are represented as mean ± SEM. Densitometric quantification of striatal TH^+ ^staining was done by a blinded observer.

### Laser capture microdissection (LCM)

Twelve-week-old male C57BL/6 mice were euthanized using CO_2 _asphyxiation. The brains were removed and immediately frozen in isopentane on dry ice. Brains were then stored at -80°C until analysis. Brains were then cut on a cryostat and 10 μm coronal sections were mounted on uncoated slides and stored at -80°C. Slides were allowed to briefly reach room temperature and were stained for TH using a rapid staining protocol to minimize mRNA degradation. Briefly, slides were fixed in acetone (40 seconds), washed briefly 3 times in phosphate buffer (PB; pH = 7.4), followed by a 5 min incubation with a mouse-anti-TH antibody (Sigma, 1:1000) diluted in PB. Slides were washed 3 times with PB, followed by 5 minute incubation with a Cy3-conjugated goat anti-mouse IgG (Jackson Labs, 1:500) diluted in PB. Slides were washed twice in PB and then dehydrated in ethanol (50%, 70%, 95%, and 100%), (10 seconds each) and delipidated in xylene (40 seconds). Slides were then allowed to air dry. All procedures were carried out in an RNAse-free environment.

Tyrosine hydroxylase positive cells of the SNpc and VTA were visualized and dissected using a PixCell laser capture microscope under a 20× objective with an infrared diode laser (Arcturus Engineering, Santa Clara, CA). Approximately 500 TH positive cells per animal from the SNpc or VTA we isolated on a single LCM HS cap (Arcturuss Arcturus Engineering, Santa Clara, CA). RNAs were isolated using a PicoPure RNA Isolation kit (Arcturus, Mountain View, CA, USA) according to manufacturer's guidelines, and subjected to DNAse treatment (Qiagen, Valencia, CA, USA).

### Real-time RT-PCR

Atgr2, TH and GAPDH mRNA expression profiles were done by quantitative real-time PCR on TH^+ ^neurons from the SNpc or VTA. Primers used are listed in table 4. The primers (18–22 mer) were designed using Primer3 [62]. These primer sets were designed to amplify small amplicons for candidate mRNAs ranging from 100–300 bp in size. First-strand cDNA synthesis was carried out on mRNA extracted with SuperscriptTM first-strand synthesis kit (Invitrogen Carlsbad, CA) according to the manufacturer's specifications. Real-time RT-PCR was carried out in a 96 well plate using a MyiQ iCycler (BioRad, Hercules, CA), and SYBR Green PCR Master Mix (Applied Biosystems, Foster City, CA). A concentration curve with known concentrations of whole brain cDNA extracts from 12-week-old male C57BL/6 mice was used to calculate standard curves and quantitate the products. The final concentration of each transcript was calculated using the MyiQ2.0 software provided by BioRad.

**Table 4:** List of primers used for Real Time RT-PCR.

Atgr2	accaatcggtcatctaccctt	left
	ggcaatgaggatagacaagcc	right
GAPDH	tggtgaagcaggcatctgag	left
	tgctgttgaagtcgcaggag	right
TH	ttctgaaggaacggactgg	left
	ggcatgacggatgactgtg	right

### Western immunoblot analysis

The expression of AT_1 _and AT_2 _receptors was examined in an immortalized mesencephalic dopaminergic cell line, N27 [63]. N27 cells were thawed into RPMI 1640 media containing 10% FBS, 2 mM glutamine, 100 U/ml penicillin and 100 μg/ml streptomycin. Cells were collected in lysis buffer (Cell Signaling Technology, Beverly, MA) with 1% SDS. After sonication, the lysate was centrifuged at 10,000 × g for 45 min at 4°C, and the proteins in the supernatant were separated on 4–12% Bis-Tris polyacrylamide gel. After transfer to PVDF membranes, the proteins were probed for AT_1 _receptors (Santa Cruz polyclonal sc-1173 or Abcam polyclonal ab 18801, 1:500 dilution), AT_2 _receptors (Santa Cruz polyclonal sc-9040 or Abcam polyclonal ab 19134, 1:500 dilution). Secondary, alkaline phosphatase-conjugated anti-rabbit (1:5000; Sigma) and anti-mouse (1:10,000, Jackson ImmunoResearch) antibodies were followed by Lumi-Phos (Pierce) for chemiluminescence detection. Blots were stripped and reprobed for β-actin (Sigma, 1:10,000) to confirm equal protein loading on the gel.

### Statistical analysis

Data were analyzed using Statistica software. Statistical significance was determined by one-way ANOVA followed by post-hoc Newman-Keuls multiple comparison tests. Differences in mean values were considered significant at p ≤ 0.05. All data were obtained from at least three independent experiments and are represented as a mean ± SEM.

## Abbreviations

ACE, angiotensin converting enzyme; Ang II, angiotensin II; ANOVA, analysis of variance; AT_1 _receptor, Ang II type 1 receptor; Agtr2, gene encoding AT_2 _receptor; BP, blood pressure; CE, coefficient of error; CV, coefficient of variance; DA, dopamine or dopaminergic; DAT, dopamine transporter; E15, embryonic day 15; GDNF, glial cell line-derived neurotrophic factor; GFAP, glial fibrillary acidic protein; GPCR, G-protein coupled receptor; HBSS, Hank's buffered salt solution; LCM, laser capture microdissection; MANF, mesencephalic astrocytes-derived neurotrophic factor; MAP2, microtubule-associated protein 2; MPTP, 1-methyl-4-phenyl-1,2,3,6-tetrahydropyridine; MPP+, 1-methyl-4-phenylpyridinium; NeuN, neuronal nuclear protein; NIH, National Institutes of Health; PD, Parkinson's disease; PKC, protein kinase C; PLA2, phospholipase 2; RAS, renin-angiotensin-system; RT-PCR, reverse transcriptase polymerase chain reaction; SNpc, substantia nigra pars compacta; TH, tyrosine hydroxylase; VM, ventral mesencephalon; VTA, ventral tagmental area.

## Competing interests

The author(s) declare that they have no competing interests.

## Authors' contributions

All authors read and approved the final manuscript. TNG, WMZ and JAW designed the research. TNG and WMZ wrote the paper. TNG, SMJ, FAA, BRH, LDS, AMP, JS and VVJ performed the research.
